# Transcutaneous auricular vagus nerve stimulation (taVNS) for the treatment of pediatric nephrotic syndrome: a pilot study

**DOI:** 10.1186/s42234-021-00084-6

**Published:** 2022-01-26

**Authors:** Kumail Merchant, Stavros Zanos, Timir Datta-Chaudhuri, Clifford S. Deutschman, Christine B. Sethna

**Affiliations:** 1 Cohen Children’s Medical Center of New York, New Hyde Park, United States NY; 2 The Feinstein Institutes for Medical Research, Manhasset, United States NY

**Keywords:** Vagus nerve stimulation, taVNS, Nephrotic syndrome, Children, Minimal change disease, Focal segmental glomerular sclerosis

## Abstract

**Background:**

Children with frequently relapsing nephrotic syndrome (FRNS) and steroid resistant nephrotic syndrome (SRNS) are exposed to immunosuppressant medications with adverse side effects and variable efficacy. Transcutaneous auricular vagus nerve stimulation (taVNS) modulates the immune system via the inflammatory reflex and has become a therapy of interest for treating immune-mediated illnesses.

**Methods:**

An open-label, pilot study of tavNS for five minutes daily for 26 weeks via a TENS 7000 unit was conducted.

**Results:**

Three FRNS participants and 4 SRNS participants had a mean age of 9.5±4.2 years (range 4 to 17). Those with FRNS remained relapse-free during the study period; two participants continued treatment and remained in remission for 15 and 21 months, respectively. Three SRNS participants experienced a reduction in first morning UPC (mean of 42%, range 25-76%). Although UPC decreased (13.7%) in one SRNS participant with congenital nephrotic syndrome, UPC remained in nephrotic range. All but one participant (non-compliant with treatment) experienced a reduction in TNF (7.33pg/mL vs. 5.46pg/mL, *p*=0.03). No adverse events or side effects were reported.

**Conclusions:**

taVNS was associated with clinical remission in FRNS and moderately reduced proteinuria in non-congenital SRNS. Further study of taVNS as a treatment for nephrotic syndrome in children is warranted.

ClinicalTrials.gov Identifier: NCT04169776, Registered November 20, 2019, https://clinicaltrials.gov/ct2/show/NCT04169776.

## Background

Nephrotic syndrome, the most common glomerular disease affecting children, is characterized by damage to podocytes in the kidney resulting in protein loss, edema and dyslipidemia. Although most children respond to steroid therapy, up to 50% develop frequently relapsing nephrotic syndrome (FRNS). Even more concerning, 10-20% develop steroid resistant nephrotic syndrome (SRNS) and 36-50% of these children progress to kidney failure (Chapter 2011). Children with FRNS and SRNS are exposed to prolonged courses of immunosuppressant medications. Given the adverse side effects and variable efficacy of these medications (Zhang et al. 2016); (Alfakeekh et al. 2019), novel and safe therapies to treat nephrotic syndrome are needed.

Although the etiology of nephrotic syndrome is not completely understood, immune system dysregulation is thought to contribute to disease pathogenesis (Youssef et al. 2015). It has been suggested that podocyte damage may be mediated by cytokines, T regulatory cells and/or circulating factors (i.e. autoantibodies) (Youssef et al. 2015); (Gomez-Chiarri et al. 1996); (Petrovic-Djergovic et al. 2015); (Kaneko et al. 2015); (Lai et al. 2007); (Kanai et al. 2010); (Araya et al. 2006); (Kaneko et al. 2002); (Bagga et al. 2007); (Dotsch et al. 2008); (Kimata et al. 2013). Electrical stimulation of the vagus nerve has been shown to activate the inflammatory reflex, which alters splenocyte cytokine release, B cell migration and antibody formation (Andersson and Tracey 2012); (Tracey 2002). Immunomodulation of the vagus nerve and spleen has been shown to reduce immune markers such as tumor necrosis factor-alpha (TNF) and confer protection from ischemia-reperfusion injury in the kidney of mice (Inoue et al. 2016); (Gigliotti et al. 2015); (Gigliotti et al. 2013); (Tanaka et al. 2021).

taVNS is a non-invasive technique that can engage the inflammatory reflex by electrically stimulating the auricular branch of the vagus nerve that innervates the cymba concha in the ear (Butt et al. 2020); (Peuker and Filler 2002); (al. 2021). Recent studies in adults have demonstrated the efficacy of taVNS in treating immune-mediated diseases such as rheumatoid arthritis and systemic lupus erythematous (SLE) (Aranow et al. 2020); (Koopman et al. 2016); (D’Haens et al. 2016); (Bonaz 2018); (Bonaz et al. 2016); (Addorisio et al. 2019). These studies, coupled with evidence from animal models (Inoue et al. 2016); (Meregnani et al. 2011); (Borovikova et al. 2000); (Huston et al. 2007); (Guarini et al. 2003); (Levy et al. 2012); (Levine et al. 2014); (Costes et al. 2014); (van Westerloo et al. 2006); (Ma et al. 2019); (Mueller et al. 2011); (https://fimr.northwell.edu/biostatRMS/ n.d), suggest that there may be a role for taVNS in the treatment of nephrotic syndrome in children. The objective of this pilot study was to evaluate taVNS for the treatment of nephrotic syndrome in children.

## Methods

### Study design and population

An open-label, pilot study of taVNS therapy in children with nephrotic syndrome was conducted at a single tertiary pediatric hospital in New York from 2019 to 2021. The study was approved by the Institutional Review Board of Northwell Health. Consent from a guardian/parent of all participants and assent from children ≥ 7 years were obtained prior to any study procedures. Children aged 3-17 years diagnosed with minimal change disease (MCD) or focal segmental glomerulosclerosis (FSGS) who were classified as either FRNS or SRNS and who had an estimated glomerular filtration rate (eGFR) ≥30 ml/min/1.73 m^2^were eligible to participate. Those with a known history of an inflammatory condition (e.g. systemic lupus erythematosis), history of cardiac disease (e.g. arrhythmias, structural/functional abnormalities), those with an implantable electronic device or who were pregnant were excluded from the study. FRNS was defined as steroid-responsive nephrotic syndrome with at least two relapses in the six months prior to enrollment (KDIGO. 2012); (KDIGO. 2020). FRNS participants on standing immunosuppression treatment (e.g. tacrolimus, mycophenolate mofetil) were not eligible. Participants with previous exposure to immunosuppression (besides corticosteroids) were eligible after stopping the product for three months or, for those previously exposed to rituximab, once B cells were replete. Participants had to be off of corticosteroids for a minimum of 14 days prior to enrollment to ensure that they were not steroid dependent and to minimize the effect of steroids on immune markers. SRNS was defined by the inability to achieve remission (urine protein:creatinine [UPC] <0.2) by four weeks of steroid therapy (Mekahli et al. 2009); (Paik et al. 2007); (Chapter 2011). All participants with SRNS were required to be on a stable regimen of medications for at least three months prior to enrollment. Doses of the immunosuppressant medications and angiotensin converting enzyme inhibitors (ACEi)/angiotensin receptor blockers (ARBs) were not changed during the treatment period with the exception of dose changes for calcineurin inhibitors to maintain a pre-specified therapeutic level. SRNS participants with previous exposure to rituximab were eligible once B cells were replete.

### Intervention

**Fig. 1:**
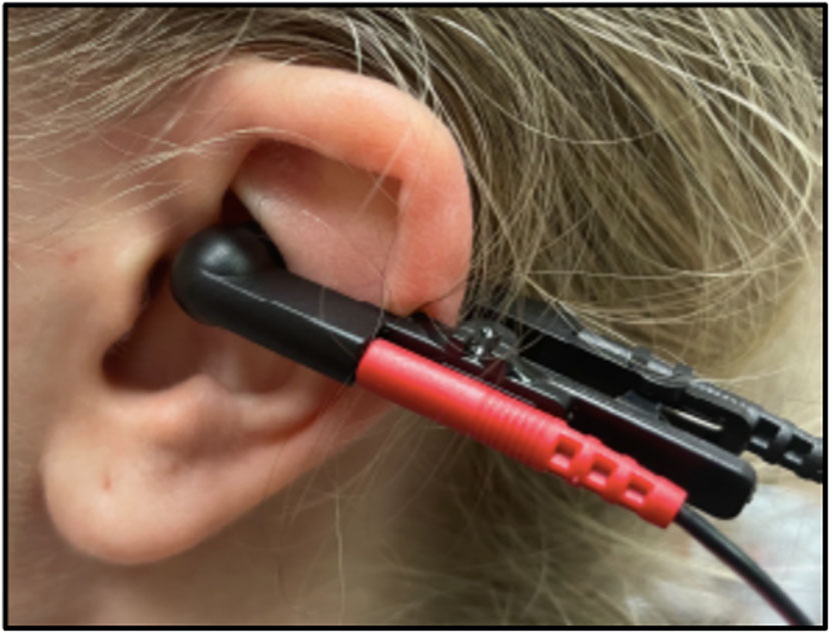
Ear-clip at the left cymba concha

A commercially available transcutaneous electrical nerve stimulation (TENS) unit (Roscoe Medical TENS 7000) was used to deliver electrical stimulation to the auricular branch of the vagus nerve via the left ear cymba concha with custom-made ear clips and electrode gel (Fig. 1) (Badran et al. 2019). taVNS was performed on the left side to reduce the potential for cardiac side effects including bradycardia (Redgrave et al. 2018). The device was set to a frequency of 30 Hz with individual pulse widths of 300 µs, and pulse amplitude intensity was adjusted to the participant’s tolerance to a maximum of 2.5 out of 10 on the full scale of the stimulator. Parents/guardians were trained to perform taVNS therapy on their child at home once a day for five minutes for 26 weeks. Parents/guardians were required to demonstrate correct taVNS usage at enrollment and monthly at each follow up visit, which alternated in person and virtually. Device settings, treatment length, and ear clip design were chosen based upon our previous findings (Aranow et al. 2020) and a survey of the literature (Aranow et al. 2021). The study period of 26 weeks was chosen based on the definition of FRNS using a 6-month period and prior clinical trials (Wang et al. 2018). Additionally, 26 weeks is a sufficient time to evaluate an improvement in proteinuria in patients with SRNS.

### Study outcomes

The primary outcome for the FRNS group was the number of relapses during the study period. The primary outcome for the SRNS group was percent change in the first morning UPC from baseline to 26 weeks. Changes in serum cytokines from baseline to 26 weeks were also measured. Additionally, feasibility outcomes such as recruitment rate, drop out rate, adherence, safety and patient satisfaction were monitored.

### Study procedures

The medical record was abstracted for the following variables: age, race, sex, age at diagnosis of nephrotic syndrome, type of nephrotic syndrome, previous response to treatment, number of previous nephrotic syndrome relapses, previous immunosuppressive therapies, and current medications.

Blood and urine samples were collected at baseline as well as every eight weeks during the study period. Complete metabolic panel and serum cytokines were measured. Cytokines were quantified using the Human High Sensitivity T-Cell Discovery Array 14-Plex (Eve Technologies Corp, Calgary, AB, Canada). The multiplex assay was performed using the Bio-Plex™ 200 system (Bio-Rad Laboratories, Inc., Hercules, CA, USA), and a Milliplex Human High Sensitivity T-Cell panel (Millipore, St. Charles, MO, USA) according to their protocol. The assay consisted of GM-CSF, IFNγ, IL-1β, IL-2, IL-4, IL-5, IL-6, IL-8, IL-10, IL-12 (p70), IL-13, IL-17 A, IL-23, and TNF. Assay sensitivities of these markers range from 0.11 to 3.25 pg/mL.

FRNS participants performed home daily testing of first morning urine for protein utilizing protein urine testing strips as per usual standard of care. Home urine protein >2+ for three or more consecutive days indicated nephrotic syndrome relapse (KDIGO. 2012); (KDIGO. 2020). A first morning UPC was sent to confirm relapses. For the SRNS group, baseline proteinuria was defined by a three-month average first morning UPC; first morning UPC was then measured every eight weeks.

Due to the concern for bradycardia, heart rate was monitored by a commercially available pulse oximeter with heart rate monitoring feature (Zacurate 500BL) during and for 1 min following each taVNS treatment. Participants were provided with normal heart rate ranges for age and were instructed to stop therapy immediately if heart rate fell above or below the normal range for age (Duff et al. 2019). Additionally, participants were instructed to stop therapy immediately for any symptoms of bradycardia, including lightheadedness, syncope or shortness of breath. Parents/guardians were instructed to keep a log of the time that the taVNS therapy was performed each day, along with concurrent heart rate during the therapy, and any issues/symptoms that were experienced during the treatment. Side effects and adverse events were monitored via survey at each study visit.

Feasibility measures including recruitment rate, drop out rate, adherence and safety were monitored. At the conclusion of the study, parents/guardians were asked the following questions, which were rated on a 5-point Likert scale (strongly agree, agree, neutral, disagree, strongly disagree): (1) Treatment with taVNS was too much of a burden for my child, and (2) I would prefer to use taVNS over starting a new immunosuppressive medication for the treatment of nephrotic syndrome for my child.

### Statistical analysis

Sample size was chosen based on feasibility and no formal sample size calculations were performed. Descriptive statistics were used in the analysis of this pilot study and cytokines were analyzed via Wilcoxon signed rank test. STATA 16 (STATA Corp LLC) statistical package was utilized.

## Results and Discussion

Seven participants, including three children with FRNS and four children with SRNS, were enrolled in the study. The participants were a mean age of 9.5±4.2 years (range 4 to 17), 57% were male, 28.5% identified as Black and 28.5% identified as Hispanic (See Table 1). FRNS participants each had two relapses in the six months prior to enrollment and 3-4 relapses in the previous 12 months. The mean duration of nephrotic syndrome diagnosis for participants with FRNS was 3±1.8 years. Two of the three participants were previously treated with mycophenolate mofetil. Among the SRNS group, three participants had biopsy-confirmed FSGS and one participant had congenital nephrotic syndrome with a genetic mutation in NPHS2. The mean duration of FSGS diagnosis was 3.3±0.55 years. Those with FSGS were on stable doses of tacrolimus and enalapril at the time of enrollment; all had previous corticosteroid exposure. The child with congenital nephrotic syndrome had a disease duration of 12 years and was on a stable regimen of enalapril and losartan at the time of enrollment (with previous exposure to corticosteroids and tacrolimus).

**Table 1:** Demographic and Clinical Measures of Study Participants

ID	Diagnosis	Age	Sex	Duration of disease (years)	Previous Medications	Change in UPC	Serum albumin (mg/dl)			# Relapses in previous 12 months	# Relapses during taVNS study
							Baseline		6 months		
001	FRNS	4	M	1.5	-	-	-			3	0
002		8	M	5	MMF	-	-			4	0
003		8	M	4	MMF	-	-			4	0
004	SRNS: FSGS	7	M	2.7	tacrolimus, enalapril, prednisolone	-76%	3.3	3.3		-	-
005		11	F	3.8	tacrolimus, enalapril, prednisolone	-36%	4.7	4.8		-	-
006		17	F	3.3	tacrolimus, enalapril, prednisolone	-25%	4.5	4.6		-	-
007	SRNS: Congenital Nephrotic	12	F	12	tacrolimus, enalapril, losartan prednisolone	-13.7%	2.5	2.4		-	-

FRNS- frequently relapsing nephrotic syndrome; SRNS- steroid resistant nephrotic syndrome; FSGS- focal segmental glomerulosclerosis; M- male; F- female; MMF- mycophenolate mofetil; UPC- urine protein:creatinine; taVNS- transcutaneous auricular vagus nerve stimulation

Among three children with FRNS, all remained relapse free during the 26-week study period. Participant 002 reported missing treatment for a period of 4 days, during which time the child began to have +2 protein on urine dip. After resuming treatment, the urine protein cleared, and the child remained in remission. Participants 001 and 002 continued treatment with taVNS beyond the study period and both have remained in remission (total 21 and 15 months, respectively), despite having upper respiratory tract infections (usual triggers). Participant 003 stopped taVNS after the study period and relapsed twice in the following 6 months. While on taVNS, all three participants had the longest relapse-free period since their nephrotic syndrome diagnosis.

**Fig. 2:**
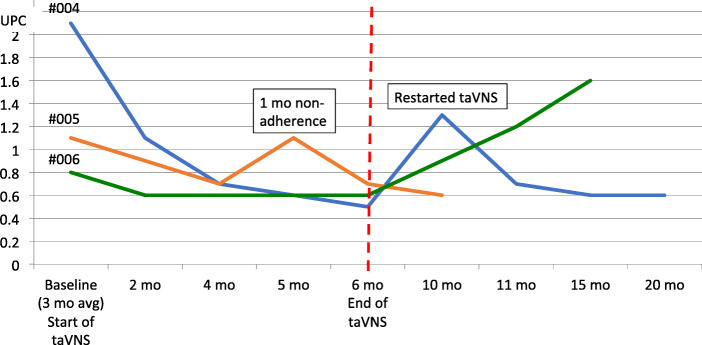
Urine protein:creatinine change in steroid resistant nephrotic syndrome participants during the study and follow-up periods

Of the participants in the SRNS group, there was a 25-76% reduction in proteinuria compared to baseline UPC in the three children with FSGS. See Fig. 2. Participant 004 who had nephrotic-range proteinuria for over 2.5 years, started with a baseline average UPC of 2.1, which decreased to 0.5 by the end of 26 weeks of taVNS. After discontinuing taVNS, the UPC increased to 1.3. taVNS was restarted and the UPC then decreased to 0.6, which was sustained at 20 months. Participant 006 also had an increase in proteinuria after discontinuation of taVNS. Although there was a demonstrated 13.7% reduction in proteinuria (UPC 5.1 to 4.1), Participant 007 with congenital nephrotic syndrome continued to have nephrotic range proteinuria.

### Cytokine analysis

**Fig. 3:**
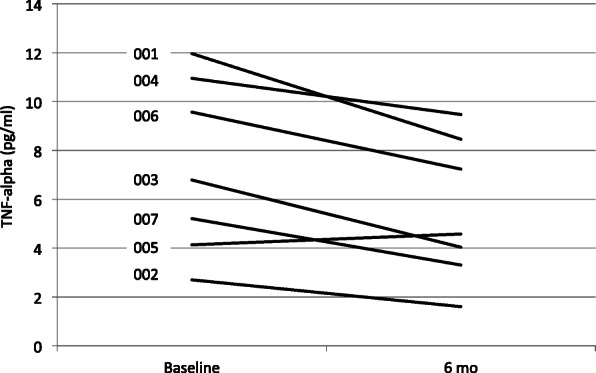
Change in TNF levels from baseline to 26 weeks while on transcutaneous vagus nerve stimulation therapy

Overall, there was a statistically significant decrease in the participants’ serum TNF level from baseline to the end of the study period (7.33 pg/mL vs. 5.46 pg/mL, *p*=0.03). See Fig. 3. Individually, all but one of the participants experienced a reduction in TNF level; the exception was Participant #005, who was noncompliant with taVNS treatments in the month prior to completion of the study (TNF decreased from baseline 4.13 pg/mL to 3.78 pg/mL at the previous study visit while the participant was compliant with the treatment protocol). There were no other significant changes noted in any of the other measured cytokines.

### Feasibility outcomes

All eligible children that were approached for study participation were successfully enrolled in the study. There were no dropouts; all participants completed the full 26-week study duration. There were no reported adverse events or side effects by any participant during the study period. Adherence to the taVNS procedures was overall very good, with the exception of the one participant who was non-adherent for the last month of the study period. There was a malfunction of the ear piece for this participant, but the parent did not report the issue until the last study visit. Parents/guardians overall disagreed with the statement that taVNS was too burdensome for their child (2 strongly disagree, 4 disagree, 1 neutral). They overall agreed with the statement that they would prefer to use taVNS over adding immunosuppressive medications (3 strongly agree, 4 agree).

Results from this pilot study suggest that taVNS is a non-invasive, steroid-sparing therapy for nephrotic syndrome in children. taVNS therapy for 26 weeks was associated with the prevention of nephrotic syndrome relapses in FRNS and reduction of proteinuria in non-congenital SRNS, and the response coincided with a significant decrease in TNF. Given that congenital nephrotic syndrome is a genetic, non-immune-mediated form of nephrotic syndrome, we did not expect the participant with a podocin mutation to respond to taVNS. Post-study follow-up demonstrated durability of results in participants that continued with daily taVNS treatment, whereas relapse/increased proteinuria occurred in those that discontinued therapy.

Surgically-implanted vagus nerve stimulation (VNS) was FDA approved for epilepsy and depression in the late 1990 s. Since that time, the inflammatory reflex of the vagus nerve has become a target of interest for treating chronic immune-mediated illnesses with taVNS. Recently, Marsal et al. demonstrated that daily taVNS resulted in significant decreases in disease activity scores after 12 weeks in 30 patients with moderate to severe rheumatoid arthritis (Marsal et al. 2021). Another study of 16 rheumatoid arthritis patients with high disease activity had lower activity scores after 4 days of taVNS (Drewes et al. 2021). Additionally, a randomized double blind study of 18 patients with systemic lupus erythematosis comparing 5 min of taVNS vs. sham for four days demonstrated improvement in pain, fatigue and global health scores at day 12 in the treatment group (Aranow et al. 2020). Similar to these studies, the reduction in nephrotic syndrome relapses in participants with FRNS and the reduction in proteinuria levels in participants with SRNS are very promising findings of the current study. Patients with nephrotic syndrome are often exposed to various immunomodulating therapies with a myriad of adverse effects combined with the variability and unpredictability of the disease process. If the hypothesis that activation of the inflammatory reflex via taVNS limits the immune response, attenuates relapses and proteinuria, and reduces reliance on immunosuppressant medications in children with nephrotic syndrome is proven to be correct, then this will lead to a paradigm shift in the treatment of nephrotic syndrome.

We found that levels of TNF, a known pro-inflammatory cytokine, were significantly decreased over the study period in this population. These results are consistent with findings in a pilot study of healthy adults that demonstrated a decrease in endotoxin-induced whole blood TNF by 80%, IL-6 by 73%, and IL-1β by 50% as compared to pre-treatment levels after auricular stimulation of the cymba concha for 2-5 minutes (Addorisio et al. 2019). It has been hypothesized that podocyte damage in nephrotic syndrome may result from dysfunctional T cells that release circulating factors including pro-inflammatory cytokines such as TNF. In a small pediatric study, TNF was shown to be higher during times of relapse (mean 4.11 pg/ml) and lower during remission (2.14 pg/ml) for steroid sensitive nephrotic syndrome, while there was no significant difference in TNF for SRNS children who remained protienuric after treatment (6.13 vs. 5.67 ng/ml) (Weisbach and GBea 2017). Further, genomic studies have found polymorphisms in TNF associated with the susceptibility for nephrotic syndrome and transcriptomic studies have shown activation of TNF in children and adults with nephrotic syndrome (Mariani LESea n.d); (Xiao 2020). In an animal model, rats infused with TNF developed proteinuria; however, the exact mechanism by which cytokines result in proteinuria is unclear (Lai et al. 2007).

Although the demonstrated decrease in TNF with taVNS in this study suggests a possible immune-mediated mechanism, the mechanisms by which taVNS interacts with the kidney in nephrotic syndrome are not known. Future mechanistic studies in animals are warranted. While ischemia-reperfusion injury of the kidney is a different disease process from nephrotic syndrome, work by Tanaka et al. in a mouse model treated with VNS provides some insight into a possible mechanism of how VNS exerts its effects on the kidney. The group mapped out two neural pathways involved in the neuro-immune protection of the kidneys with VNS (Tanaka et al. 2021). Using optogenetics to selectively stimulate nerve fibers, they found that anterograde stimulation of both efferent and afferent vagus nerves resulted in kidney protection, and the protection was mediated by the spleen. They showed that the vagus efferent pathway exerted its effect through activating the cholinergic anti-Inflammatory pathway. They found that the vagus afferent pathway protected the kidneys by activating the vago-sympathetic pathway via the splenic nerve to the spleen. This is an interesting finding, as taVNS triggers afferent vagus nerves.

An important aspect of this study was to determine the feasibility of daily taVNS therapy at home over an extended period (26 weeks). There were no reported side effects or adverse events. Participants and their family members endorsed that study protocol was not burdensome and daily treatments at home was feasible. This is important moving forward with future investigations as being able to utilize this device at home expands the utility of taVNS in the treatment of pediatric diseases.

The study has important limitations to consider. As this was an open-label pilot study in a small group of participants without a control group or randomization, the results must be interpreted with caution. A larger, randomized clinical trial would be needed to prove that taVNS treatment in nephrotic syndrome is safe and effective. Nephrotic syndrome is also a waxing and waning disease, so it is difficult to attribute the lack of relapses directly to taVNS therapy without a control group. However, using the participants as their own historical controls demonstrated that those with FRNS remained relapse-free for the longest period since their disease diagnosis and relapses did not occur with typical triggers such as upper respiratory tract infections. And, those with SRNS had a reduction in proteinuria compared to their historical values, especially the participant who had a reduction in proteinuria to near complete remission after having nephrotic range proteinuria for over 2.5 years. Furthermore, the optimal prescription of taVNS for use in nephrotic syndrome, such as the duration, timing and dose of treatment, are also unknown.

## Conclusions

To our knowledge, this is the first intervention study with the aim of modulating vagus nerve activity as an approach to treat nephrotic syndrome in humans. taVNS has the potential to provide a non-pharmacologic option for the treatment of nephrotic syndrome in children and is a steroid-sparing therapy that can possibly prevent the deleterious side effects of immunosuppressant medications in children with nephrotic syndrome. Our data indicate that taVNS is a promising, novel, non-pharmacologic, non-invasive, steroid-sparing approach to the treatment of nephrotic syndrome in children. We believe that the findings are sufficient to justify a randomized clinical trial to investigate the efficacy and safety of taVNS in the treatment of nephrotic syndrome in children.

## Data Availability

Available upon request.
